# Association between Gut Microbiota and Digestive System Cancers: A Bidirectional Two-Sample Mendelian Randomization Study

**DOI:** 10.3390/nu15132937

**Published:** 2023-06-28

**Authors:** Ning Xie, Ziwei Wang, Qiuai Shu, Xiru Liang, Jinhai Wang, Kaichun Wu, Yongzhan Nie, Yongquan Shi, Daiming Fan, Jian Wu

**Affiliations:** 1 Department of Gastroenterology, The Second Affiliated Hospital, Xi’an Jiaotong University, Xi’an 710049, China; wzw723@stu.xjtu.edu.cn (Z.W.); xjsqa912722614@stu.xjtu.edu.cn (Q.S.); lxr857439646@stu.xjtu.edu.cn (X.L.); jinhaiwang@hotmail.com (J.W.); 2 Bioinspired Engineering and Biomechanics Center (BEBC), Xi’an Jiaotong University, Xi’an 710049, China; 3 National Clinical Research Center for Digestive Diseases, State Key Laboratory of Cancer Biology, Xijing Hospital of Digestive Diseases, Air Force Medical University, Xi’an 710032, China; kaicwu@fmmu.edu.cn (K.W.); yongznie@fmmu.edu.cn (Y.N.); shiyquan@fmmu.edu.cn (Y.S.); fandaim@fmmu.edu.cn (D.F.)

**Keywords:** causality, digestive system cancers, gut microbiota, Mendelian randomization

## Abstract

Accumulating evidence indicates that gut microbiota closely correlates with the tumorigenesis of digestive system cancers (DSCs). However, whether the causality between gut microbiota and DSCs exists is unknown. Genome-wide association study (GWAS) summary statistics for gut microbiota and DSCs and the bidirectional two-sample Mendelian randomization (MR) analysis were utilized to assess the causality between gut microbiota and DSCs. Sensitivity analyses were performed to evaluate the robustness of our results. We found that the *genus Eggerthella* (OR = 0.464, 95%CI: 0.27 to 0.796, *p* = 0.005) was negatively associated with the risk of gastric cancer. The genetically predicted *genus Lachnospiraceae FCS020 group* (OR = 0.607, 95%CI: 0.439 to 0.84, *p* = 0.003) correlated with a lower risk of colorectal cancer, and *genus Turicibacter* (OR = 0.271, 95%CI: 0.109 to 0.676, *p* = 0.005) was a protective factor for liver cancer. In the reverse MR, DSCs regulated the relative abundance of specific strains of gut microbiota. We comprehensively screened the association between gut microbiota and DSCs using a bidirectional two-sample MR analysis and identified the causality between several microbial taxa and DSCs. Our discoveries are beneficial for the development of novel microbial markers and microbiota-modifying therapeutics for DSC patients.

## 1. Introduction

Digestive system cancers (DSCs) comprise gastrointestinal cancers (esophageal cancer, gastric cancer, small intestinal cancer, colorectal cancer) and hepatobiliary–pancreatic cancers (liver cancer, gallbladder cancer, biliary duct cancer, pancreatic cancer). In general, DSCs are the most commonly diagnosed cancers and the leading cause of cancer deaths throughout the world, as they account for >26.4% of new cancer cases and >36.3% of new cancer deaths, according to the GLOBOCAN estimates of cancer incidence and mortality in 2020 by the International Agency for Research on Cancer [1]. Hence, to effectively increase life expectancy, it is a priority for us to further elucidate the specific mechanisms underlying the occurrence and progression of DSCs. To date, growing evidence has demonstrated that gut microbiota could regulate the tumorigenesis of DSCs [2,3].

The human gastrointestinal tract is a natural habitat for trillions of microorganisms, i.e., gut microbiota, which comprises bacteria, fungi, archaea, viruses, and yeast. With the rapid advances in multi-omics and sequencing technologies in recent years, our understanding of the complex relationships between gut microbiota and various disorders (e.g., metabolic diseases, gut diseases, and multiple cancers) has been greatly facilitated [4]. Notably, cumulative findings demonstrate that gut microbes and their metabolites not only regulate the development and progression of several DSCs but also modulate the DSC patients’ responsiveness to chemotherapy, radiotherapy, molecular targeted therapy, and immunotherapy largely by affecting innate and adaptive immunity [2,5,6]. For instance, specific bacteria strains, e.g., *Fusobacterium nucleatum*, *Bacteroides fragilis*, and *Escherichia coli*, are demonstrated to be closely correlated with colorectal cancer (CRC) [7]. Furthermore, *Porphyromonas gingivalis* and *Porphyromonas asaccharolytica* could promote the onset of CRC by inducing butyrate-related cellular senescence [8]. In addition, a two-sample Mendelian randomization (MR) study reported that *Ruminococcaceae*, *Porphyromonadaceae*, and *Bacteroidetes* might be protective factors for liver cancer, and the researchers further validated the findings using a case-control study [9]. Moreover, *Clostridium* species can recruit NKT immune cells to reduce liver cancer growth and metastasis by mediating bile acid metabolism [10]. Gut microbiota and their metabolites have “double-edged sword” effects on DSC patients’ responsiveness to pharmacological treatments. On the one hand, gut microbiota contributes to the development of drug resistance in DSC patients. For example, *F. nucleatum* promotes colorectal cancer chemoresistance by activating cell autophagy [11]. On the other hand, gut microbiota could boost the therapeutic efficacy and reduce the therapy-associated toxicity or side effects for DSC patients. In mouse models, *Bifidobacterium pseudolongum* was found to enhance immune checkpoint inhibitor efficacy by producing inosine to promote Th1 transcriptional differentiation [12]. Nonetheless, the current studies did not systematically examine the relationships between gut bacteria and different DSCs. More importantly, these observational studies on the association between gut microbiota and DSCs are not capable of identifying the causal link.

As a widely accepted statistical method, MR can exploit genetic variants, i.e., single nucleotide polymorphisms (SNPs), as instrumental variables (IVs) to explore the causality between interested exposures and specific outcomes [13]. As genetic variants are subject to random allocation at conception and almost remain static after birth, an MR study is capable of largely avoiding confounding factors and reverse causality in traditional observational studies [14]. To obtain a better understanding of the role of gut bacteria in DSCs, we conducted a bidirectional two-sample MR study to systematically investigate the possible causal associations between gut bacteria and diverse DSCs.

## 2. Method

### 2.1. Study Design

**Figure 1:**
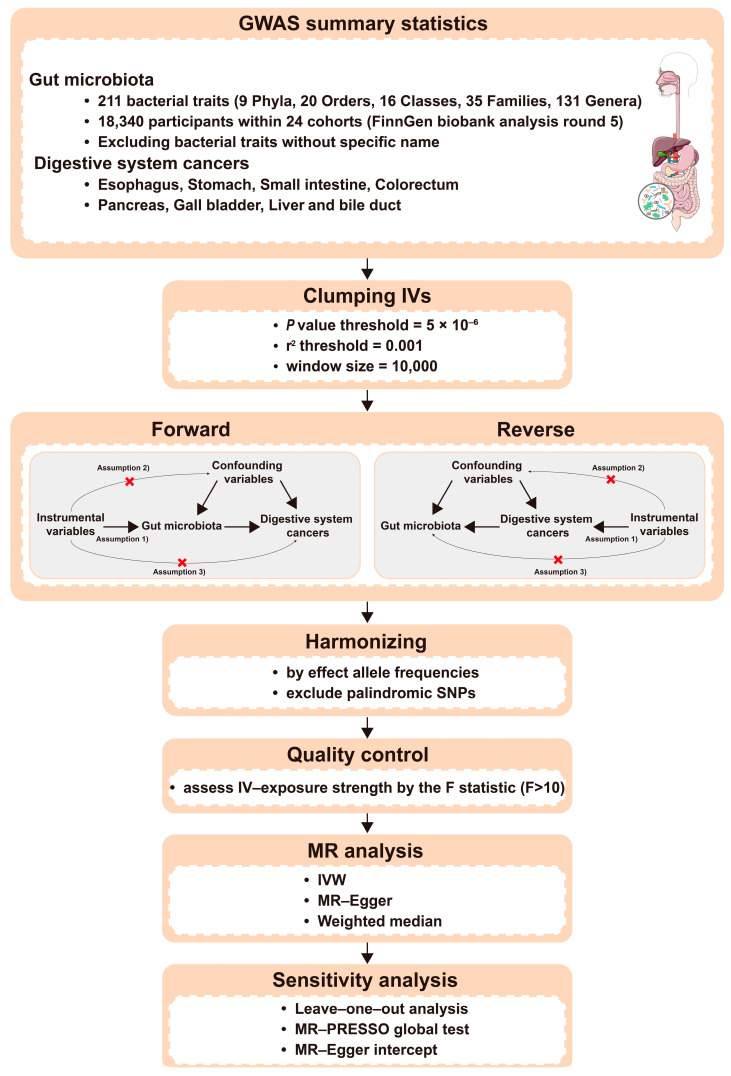
Workflow showing our two-sample bidirectional Mendelian Randomization (MR) analysis. GWAS: genome-wide association study; IVs: instrumental variables; IVW: inverse variance weighted; MR-PRESSO: Mendelian randomization pleiotropy residual sum and outlier; SNPs: single nucleotide polymorphisms.

A flowchart summarizing the bidirectional two-sample MR analysis that we used in this study is shown in Figure 1. The bidirectional two-sample MR analysis was utilized to explore the causal relationships between gut microbiota and cancers of the digestive system using the genetic variants as the IVs. Three assumptions need to be fulfilled to verify the validity of the MR analysis: (1) IVs correlate strongly with the exposure; (2) there is no correlation between IVs and any confounding variables; and (3) IVs could only affect the outcome via the exposure [15]. 

### 2.2. Data Sources and Study Population

The genome-wide association study (GWAS) statistics on gut microbiota were obtained from the IEU open GWAS database (https://gwas.mrcieu.ac.uk/, accessed on 3 October 2022), a database of 245,341,232,597 genetic associations from 42,335 GWAS summary datasets. A total of 211 bacterial traits (classified into specific phylum, class, order, family, and genus) were obtained, and the sample size was 14,306. Since 15 bacterial traits did not have specific species names, we excluded them and selected 196 bacterial traits for analysis.

We initially selected seven cancers of the digestive system including esophagus cancer, gastric cancer, small intestine cancer, colorectal cancer, pancreatic cancer, gallbladder cancer, and liver cancer. The GWAS summary data on the cancers of the digestive system noted above were accessed from the IEU open GWAS project (https://gwas.mrcieu.ac.uk/, accessed on 3 October 2022). The details of the GWAS datasets we selected are shown in Table 1. Esophageal cancer was excluded from this study since the IVs in datasets did not meet the requirements of MR analysis.

All participants in the above datasets except gallbladder cancer were of European ethnicity. Gallbladder cancer was excluded from this study due to the incompatibility of ethnicity in the dataset with other datasets. Ethical approval and consent to participate were obtained in all original studies.

### 2.3. Selection of IVs

Firstly, the SNPs in the GWAS summary data for exposures that were genetically associated with the traits with genome-wide significance (*p* < 5 × 10^−8^) were sifted out as instrumental variables. In such cases where the number of IVs was fewer than 4, the significance threshold was relaxed to 5 × 10^−6^ to avoid inaccurate results due to insufficient SNPs. Secondly, to exclude some undesirable SNPs (r^2^ > 0.001, window size < 10,000 kb), linkage disequilibrium clumping was utilized. Thirdly, the IVs strongly associated with outcomes (*p* < 5 × 10^−8^) were removed according to the third assumption of MR. Finally, harmonization of the exposure and outcome datasets was accomplished, and the palindromic SNPs with intermediate allele frequencies were removed.

The strength of genetic instruments for exposures was ensured by calculating the F statistic. The F statistics were computed using the admittedly reliable formula: F = R^2^ × [(N – 1 − k)/k] × (1 − R^2^), where R^2^ and N refer to the cumulative explained variance in the selected SNPs and sample size, respectively. R^2^ was calculated using the formula: R^2^ = 2 × MAF × (1 − MAF) × *β*^2^, where MAF refers to the minor allele frequency [13]. If F > 10, there is sufficient strength to avoid the problem of weak instrument bias in the two-sample model [14].

## 3. Statistical Analysis

The statistical analyses were performed in R 4.1.3 with the R packages “TwoSampleMR” (version 0.5.6) and “MRPRESSO” (version 1.0). *p* < 0.05 was considered statistical significance for evidence of a potential causal effect. The multiple methods for the main analyses (inverse variance weighted, weighted median, and MR Egger) and several sensitivity analyses (heterogeneity test, pleiotropy test, and leave-one-out sensitivity test) were utilized in our study [16,17]. We utilized the inverse-variance-weighted (IVW) method as the primary approach to estimate the causal effect. Weighted median and MR Egger were used as auxiliary methods. The sensitivity analyses were performed to evaluate the robustness of the findings. The heterogeneity test was performed using Cochran’s Q test. For the pleiotropy test, the MR-Egger intercept and MR-pleiotropy residual sum and outlier (MR-PRESSO) methods were used to evaluate pleiotropy. The MR-Egger intercept was used to evaluate the potential pleiotropy in the IVW model, and MR-PRESSO was used for testing horizontal pleiotropy by identifying and removing outlying instrumental variables (NbDistribution = 3000, SignifThreshold = 0.05) [18]. The leave-one-out sensitivity test was also used to evaluate the robustness of this study’s findings. The estimates were presented as odds ratio (OR) or *β* with their 95% confidence intervals (CIs) per one standard deviation (SD) increase in the exposure.

## 4. Results

### 4.1. Instrumental Variables

To analyze the effects of cancers on gut microbiota, we selected five SNPs for GC, five SNPs for small intestinal cancer, nine SNPs for CRC, three SNPs for pancreatic cancer, and two SNPs for liver cancer as instrumental variables with the genome-wide significance of *p* < 5 × 10^−6^. To analyze the effects of gut microbiota on cancers, we selected at least two and up to twelve SNPs for gut microbiota species as instrumental variables. Some analyses failed because no SNPs were left after harmonization. The F statistics for IVs indicated that the estimates were less likely to suffer weak instrumental bias (F > 10, Supplemental Table S1).

### 4.2. The Bidirectional Causal Associations between Gut Microbiota and DSCs

We utilized bidirectional MR analysis to investigate the causality between gut microbiota and five types of cancers (i.e., GC, small intestinal cancer, CRC, pancreatic cancer, and liver cancer). All the significant results are shown in Table 2 and Table 3.

#### 4.2.1. Gastric Cancer

The estimates calculated using the IVW test indicated that the genetically predicted relative abundance of *family Bacteroaceae* (OR = 0.156, 95%CI: 0.053 to 0.459, *p* = 0.001), *family Enterobacteriaceae* (OR = 0.206, 95%CI: 0.067 to 0.636, *p* = 0.006), *genus Bacteroes* (OR = 0.156, 95%CI: 0.053 to 0.459, *p* = 0.001), *genus Eggerthella* (OR = 0.464, 95%CI: 0.270 to 0.796, *p* = 0.005), *genus Lachnospira* (OR = 0.079, 95%CI: 0.008 to 0.739, *p* = 0.026), and *order Enterobacteriales* (OR = 0.206, 95%CI: 0.067 to 0.636, *p* = 0.006) was negatively associated with the risk of GC. The genetically predicted relative abundance of *genus Escherichia Shigella* (OR = 3.099, 95%CI: 1.152 to 8.338, *p* = 0.025), *genus Eubacterium fissicatena group* (OR = 1.648, 95%CI: 1.028 to 2.642, *p* = 0.038), and *genus Ruminococcaceae UCG014* (OR = 1.911, 95%CI: 1.010 to 3.615, *p* = 0.047) was positively associated with the risk of GC.

The estimates calculated using the IVW test indicated that the genetic predisposition to GC was negatively associated with the relative abundance of *class Methanobacteria* (*β* = −0.087, 95%CI: −0.161 to −0.012, *p* = 0.023), *family Methanobacteriaceae* (*β* = −0.087, 95%CI: −0.161 to −0.012, *p* = 0.023), *family Oxalobacteraceae* (*β* = −0.076, 95%CI: −0.137 to −0.015, *p* = 0.014), *genus Methanobrevibacter* (*β* = −0.109, 95%CI: −0.184 to −0.033, *p* = 0.005), *genus Oxalobacter* (*β* = −0.070, 95%CI: −0.134 to −0.006, *p* = 0.032), *order Methanobacteriales* (*β* = −0.087, 95%CI: −0.161 to −0.012, *p* = 0.023), and *phylum Euryarchaeota* (*β* = −0.074, 95%CI: −0.147 to −0.001, *p* = 0.047). The genetic predisposition to GC was positively associated with the relative abundance of *genus Dialister* (*β* = 0.070, 95%CI: 0.029 to 0.110, *p* = 0.001) and *genus Eubacterium ventriosum group* (*β* = 0.039, 95%CI: 0.003 to 0.075, *p* = 0.032).

#### 4.2.2. Small Intestinal Cancer

The estimates calculated using the IVW test indicated that the genetically predicted relative abundance of *family Clostriales vadin BB60 group* (OR = 0.422, 95%CI: 0.194 to 0.918, *p* = 0.03), *family Peptostreptococcaceae* (OR = 0.292, 95%CI: 0.093 to 0.918, *p* = 0.035), *genus Anaerofilum* (OR = 0.444, 95%CI: 0.211 to 0.936, *p* = 0.033), *genus Streptococcus* (OR = 0.266, 95%CI: 0.073 to 0.969, *p* = 0.045), and *order Lactobacillales* (OR = 0.314, 95%CI: 0.109 to 0.903, *p* = 0.032) was negatively associated with the risk of small intestinal cancer. The genetically predicted relative abundance of *genus Candatus Soleaferrea* (OR = 5.166, 95%CI: 1.171 to 22.797, *p* = 0.030) was positively associated with the risk of small intestinal cancer.

The estimates calculated using the IVW test indicated that the genetic predisposition to small intestinal cancer was negatively associated with the relative abundance of *genus Intestinibacter* (*β* = −0.033, 95%CI: −0.060 to −0.005, *p* = 0.022), *genus Lachnoclostrium* (*β* = −0.024, 95%CI: −0.044 to −0.003, *p* = 0.023), and *genus Peptococcus* (*β* = −0.051, 95%CI: −0.086 to −0.016, *p* = 0.004). The genetic predisposition to small intestinal cancer was positively associated with the relative abundance of *genus Collinsella* (*β* = 0.040, 95%CI: 0.018 to 0.063, *p* = 4.8E-04), *genus Erysipelotrichaceae UCG003* (*β* = 0.057, 95%CI: 0.001 to 0.113, *p* = 0.046), *genus Eubacterium ruminantium group* (*β* = 0.038, 95%CI: 0.005 to 0.072, *p* = 0.026), *genus Howardella* (*β* = 0.085, 95%CI: 0.031 to 0.138, *p* = 0.002), and *genus Lachnospiraceae UCG008* (*β* = 0.051, 95%CI: 0.018 to 0.084, *p* = 0.002).

#### 4.2.3. Colorectal Cancer

The estimates calculated using the IVW test indicated that the genetically predicted relative abundance of *family Clostriales vadin BB60 group* (OR = 0.751, 95%CI: 0.569 to 0.992, *p* = 0.044), *genus Lachnospiraceae FCS020 group* (OR = 0.607, 95%CI: 0.439 to 0.84, *p* = 0.003), *phylum Euryarchaeota* (OR = 0.801, 95%CI: 0.650 to 0.986, *p* = 0.036), and *phylum Proteobacteria* (OR = 0.613, 95%CI: 0.387 to 0.971, *p* = 0.037) was negatively associated with the risk of CRC.

The estimates calculated using the IVW test indicated that the genetic predisposition to CRC was negatively associated with the relative abundance of *genus Lactococcus* (*β* = −0.157, 95%CI: −0.263 to −0.051, *p* = 0.004) and *genus Marvinbryantia* (*β* = −0.081, 95%CI: −0.148 to −0.015, *p* = 0.016). The genetic predisposition to CRC was positively associated with the relative abundance of *genus Eggerthella* (*β* = 0.100, 95%CI: 0.002 to 0.197, *p* = 0.045) and *genus Eisenbergiella* (*β* = 0.106, 95%CI: 0.016 to 0.196, *p* = 0.021).

#### 4.2.4. Pancreatic Cancer

The estimates calculated using the IVW test indicated that the genetically predicted relative abundance of *genus Bilophila* (OR = 0.187, 95%CI: 0.041 to 0.855, *p* = 0.031), *p* = 0.029) and *genus Streptococcus* (OR = 0.253, 95%CI: 0.072 to 0.888, *p* = 0.032) was negatively associated with the risk of pancreatic cancer. And the genetically predicted relative abundance of *genus Ruminococcaceae UCG014* (OR = 6.101, 95%CI: 1.206 to 30.873) was positively associated with the risk of pancreatic cancer.

The estimates calculated using the IVW test indicated that the genetic predisposition to pancreatic cancer was negatively associated with the relative abundance of *genus Eubacterium ruminantium group* (*β* = −0.104, 95%CI: −0.186 to −0.022, *p* = 0.013) and *genus Lachnospiraceae NC2004 group* (*β* = −0.116, 95%CI: −0.208 to −0.025, *p* = 0.013). The genetic predisposition to pancreatic cancer was positively associated with the relative abundance of *genus Ruminococcaceae UCG014* (*β* = 0.088, 95%CI: 0.028 to 0.148, *p* = 0.004).

#### 4.2.5. Liver Cancer

The estimates calculated using the IVW test indicated that the genetically predicted relative abundance of *family Rhodospirillaceae* (OR = 0.357, 95%CI: 0.157 to 0.809, *p* = 0.014), *genus Escherichia Shigella* (OR = 0.198, 95%CI: 0.048 to 0.821, *p* = 0.026), *genus Eubacterium nodatum group* (OR = 0.382, 95%CI: 0.186 to 0.783, *p* = 0.009), *genus Family XIII AD3011 group* (OR = 0.261, 95%CI: 0.078 to 0.876, *p* = 0.030), and *genus Turicibacter* (OR = 0.271, 95%CI: 0.109 to 0.676, *p* = 0.005) was negatively associated with the risk of liver cancer. The genetically predicted relative abundance of *genus Dorea* (OR = 8.102, 95%CI: 1.643 to 39.965, *p* = 0.010), *genus Lachnospiraceae UCG004* (OR = 3.199, 95%CI: 1.059 to 9.662, *p* = 0.039), *genus Oscillibacter* (OR = 2.129, 95%CI: 1.123 to 4.035, *p* = 0.021), and *genus Paraprevotella* (OR = 1.961, 95%CI: 1.058 to 3.636, *p* = 0.032) was positively associated with the risk of liver cancer.

The estimates calculated using the IVW test indicated that the genetic predisposition to liver cancer was negatively associated with the relative abundance of *genus Butyricimonas* (*β* = −0.056, 95%CI: −0.107 to −0.005, *p* = 0.032) and *genus Eubacterium nodatum group* (*β* = −0.104, 95%CI: −0.197 to −0.01, *p* = 0.029). The genetic predisposition to liver cancer was positively associated with the relative abundance of *family Actinomycetaceae* (*β* = 0.071, 95%CI: 0.008 to 0.135, *p* = 0.027), *family Lactobacillaceae* (*β* = 0.069, 95%CI: 0.006 to 0.132, *p* = 0.033), *genus Eubacterium brachy group* (*β* = 0.087, 95%CI: 0.002 to 0.171, *p* = 0.044), *genus Lactobacillus* (*β* = 0.068, 95%CI: 0.004 to 0.132, *p* = 0.036), and *order Actinomycetales* (*β* = 0.071, 95%CI: 0.009 to 0.133, *p* = 0.024).

### 4.3. Sensitivity Analysis

**Figure 2:**
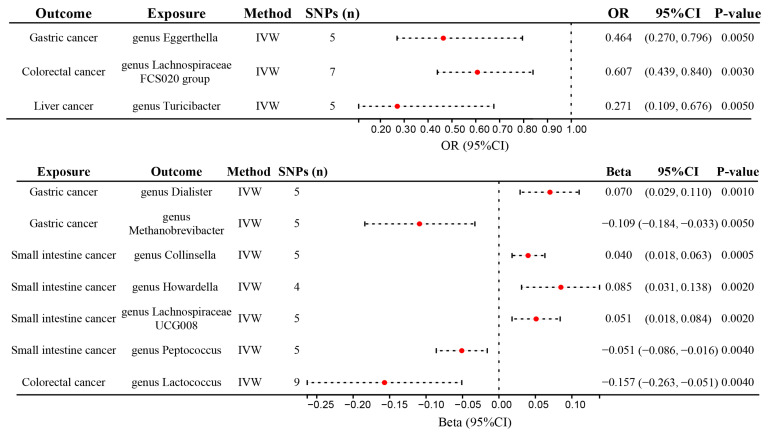
Forest plot showing the causal relationship between the genetically identified 10 microbial taxa and DSCs using the bidirectional MR analysis. DSCs, digestive system cancers; IVW, inverse variance weighted; Red dot, OR value or Beta value.

**Figure 3:**
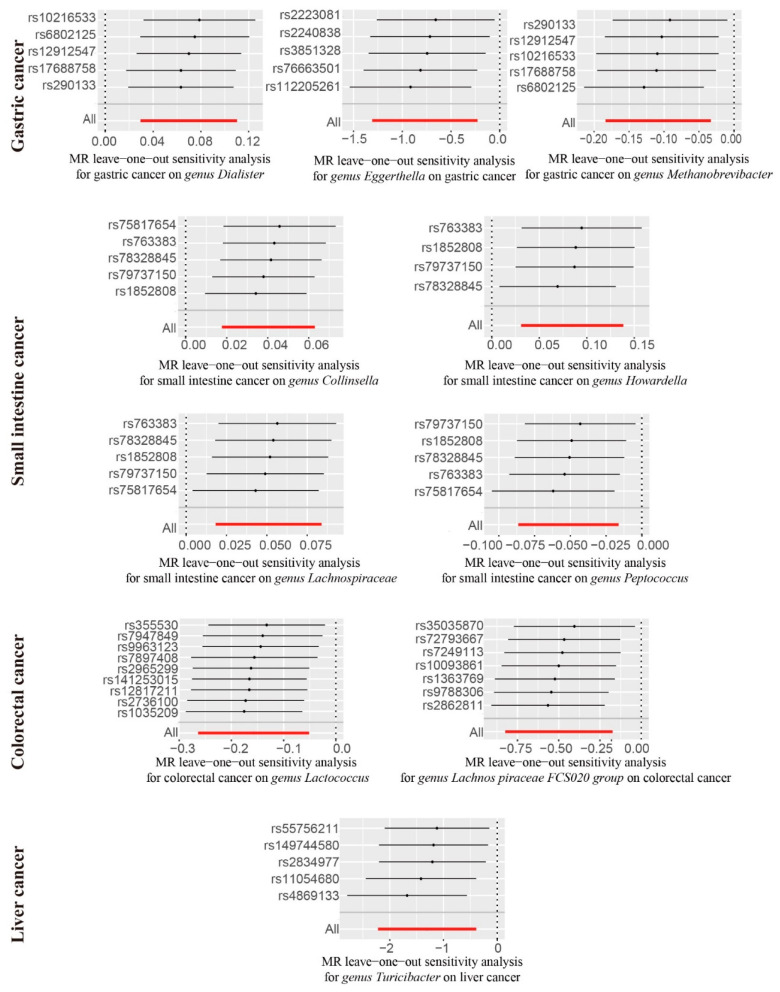
Bidirectional MR leave-one-out analysis.

We performed sensitivity analyses to evaluate the robustness of the findings for the exposures with over four SNPs as IVs. We found ten significant robust results after all the sensitivity analyses were performed (Figure 2 and Supplemental Table S2). The significant causal associations between the *genus Eggerthella* and the lower risk of GC, between the *genus Lachnospiraceae FCS020 group* and the lower risk of CRC, and between the *genus Turicibacter* and the lower risk of liver cancer were robust. Moreover, the significant causal associations between GC and the higher relative abundance of *genus Dialister* and *genus Eubacterium ventriosum group*, between small intestinal cancer and the higher relative abundance of *genus Collinsella*, *genus Howardella*, and *genus Lachnospiraceae UCG008*, between small intestinal cancer and the lower relative abundance of *genus Peptococcus*, and between CRC and the lower relative abundance of *genus Lactococcus* were robust. For these results, pleiotropic effects among the selected SNPs were not found using the MR-Egger and MR-PROSSO global tests (*p* > 0.05, Supplemental Table S2). No evidence of heterogeneity was observed between the selected IVs and cancers using Cochran’s Q test (*p* > 0.05, Supplemental Table S2). The robust results verified using the leave-one-out sensitivity test showed that removing any SNP had no effect on these results (Figure 3).

## 5. Discussion

Our study is the first attempt to systematically analyze the causal associations between gut microbiota and DSCs using a bidirectional two-sample Mendelian Randomization study. We found that the genetically increased relative abundance of specific gut microbial genera was related to the lower risk of several DSCs, i.e., *genus Eggerthella* for GC, *genus Lachnospiraceae FCS020 group* for CRC, and *genus Turicibacter* for liver cancer, respectively. Furthermore, the reverse MR analysis identified significant associations between genetic predisposition to DSCs and the relative abundance of several bacterial genera, i.e., a positive association between GC and *genus Dialister* and *genus Eubacterium ventriosum group*, a positive association between small intestinal cancer and *genus Collinsella*, *genus Howardella* and *genus Lachnospiraceae UCG008*, a negative association between small intestinal cancer and *genus Peptococcus*, and a negative association between CRC and *genus Lactococcus*.

Gut dysbiosis is widely recognized to be implicated in DSCs, and current studies have partially discovered how the dysregulation of gut microbiota promotes the occurrence and development of DSCs [19]. On the one hand, microbiota dysbiosis can induce the oncogenic transformation of host cells with direct effects on them. For instance, *campylobacter jejuni* can directly evoke DNA double-strand breaks by producing a genotoxin with DNase activity, a cytolethal-distending toxin, which aids the onset of CRC [20]. In addition, microbiota taxa such as *Helicobacter pylori* might facilitate genomic instability by inhibiting DNA mismatch repair, which contributes to gastric carcinogenesis [21]. On the other hand, gut microbiota dysregulation could shape anti-tumor immunity by exerting effects on the innate and adaptive immune responses implicated in DSCs. *Fusobacterium nucleatum* could suppress anti-tumor immunity by directly interacting with the inhibitory T-cell receptor TIGIT via FAP2 and inhibiting natural killer (NK) cell-mediated tumor killing [22,23]. Additionally, metabolites synthesized or processed by gut microbiota, e.g., short-chain fatty acids (SCFAs) and secondary bile acids (BAs), can play an immunomodulatory role. As the representative products of dietary fiber fermentation by gut microorganisms, SCFAs (e.g., butyrate, acetate, and propionate) can not only promote the generation of regulatory T cells by impeding histone deacetylase (HDAC) activity but also enhance the expansion of macrophage precursors and the function of CD8^+^ T cells by regulating cellular metabolism [24,25], which results in the paradoxical role of SCFAs in tumors [7].

Our results, for the first time, suggested that the *genus Eggerthella* had protective effects against GC. Recently, mounting evidence has jointly underlined that *Eggerthella* was associated with various diseases, such as asthma [26], multiple sclerosis [27], systemic lupus erythematosus [28], rheumatoid arthritis [29], and CRC [30]. For instance, in cigarette smoke-exposed mice, *Eggerthella lenta* might increase the bile acid metabolite, i.e., taurodeoxycholic acid (TDCA), to activate oncogenic MAPK/ERK signaling and induce gut barrier dysfunction, thus playing a protumorigenic role in CRC [30]. Since bile acid and its metabolites could influence gastric carcinogenesis [31], the *genus Eggerthella* is probably implicated in GC, but its exact role needs further exploration.

Additionally, we found that the *genus Lachnospiraceae FCS020 group* was a protective factor against CRC. Likewise, researchers found that the abundance of *genus Lachnospira*, a member of the *Lachnospiraceae family*, was reduced in CRC patients [32], and the *Lachnospiraceae FCS020 group* was negatively related to lymph node metastasis in CRC [33]. Furthermore, the *Lachnospiraceae FCS020 group* was reported to be linked with lower plasma trimethylamine N-oxide levels [34], which was a risk factor for CRC [35] and might facilitate CRC by inducing chronic inflammation, oxidative stress, and DNA damage [36]. The *Lachnospiraceae family* also may exert protective effects against CRC by enabling the immune surveillance of CD8^+^ T cells [37] and inhibiting the colonization of CRC-associated oral bacteria [38]. Although most studies report the anti-cancer effects of *Lachnospiraceae*, some studies suggest the opposite results. Secondary bile acids (BAs), including taurodeoxycholic acid and deoxycholic acid, are identified as canonical carcinogenic bile acids [30,31,39,40]. Hence, it is plausible that *Lachnospiraceae* plays a tumorigenic role in CRC as it could convert primary bile acids into secondary bile acids to increase the abundance of secondary BAs [39]. Moreover, as discussed above, SCFAs could play a dual role (pro-carcinogenic and anti-cancer effects) in the development of tumors. Considering the SCFA-producing capability of *Lachnospiraceae* and the aforementioned research results, its specific effects on CRC remain to be further elucidated.

Moreover, we found that the *genus Turicibacter* was negatively associated with the risk of liver cancer. However, the association between the *genus Turicibacter* and liver cancer has not been reported or fully defined in the published literature. *Turicibacter* has been proposed as a beneficial bacterium with anti-inflammation effects [41]. In HBV-CLD (chronic hepatitis B virus infection-associated liver diseases) patients, *Turicibacter* was inversely correlated with serum aspartate aminotransferase, total bilirubin, and direct bilirubin, which suggested its negative association with disease progression [42]. Researchers demonstrated that the *genus Turicibacter* markedly increased in NAFLD (nonalcoholic fatty liver disease) patients with significant liver fibrosis compared to those without liver fibrosis [43], but they did not prove cause and effect. Since HBV-CLD and NAFLD are significant risk factors and common causes of liver cancer, we presume that the *genus Turicibacter* may contribute to the development of liver cancer with its effects on these two liver diseases, which warrants further mechanistic studies.

Using the reverse MR analysis, we discovered that GC, small intestine cancer, and CRC were associated with the abundance of several specific gut bacterial genera. For instance, we found that GC was markedly correlated with the increased abundance of *genus Dialister* in the gut. This was supported by the published findings that the relative abundance of *Dialister* was significantly higher in GC than in other benign gastric lesions [44,45]. Additionally, *Eubacterium ventriosum group* was first identified as a GC-related microbe in our study, which was reported to be associated with a lower risk of IBD [46]. Notably, we revealed several previously unreported small intestine cancer-related bacteria. Specifically, for the first time, we identified that the *genus Collinsella, genus Howardella, and genus Lachnospiraceae UCG008* increased and that the *genus Peptococcus* decreased in small intestine cancer patients, in spite of the reported associations between *Collinsella* and anti-PD-1 efficacy [47], between *Collinsella* and oesophageal adenocarcinoma [48], between *Lachnospiraceae* and anti-PD-1 efficacy [49], between *Lachnospiraceae* and CRC [37], and between *Peptococcus* and CRC [50]. Moreover, our study showed that CRC was associated with a lower abundance of *genus Lactococcus*. However, contradictory findings about the *genus Lactococcus* in CRC exist. Researchers found that *Lactococcus lactis*, a bacterium belonging to the *genus Lactococcus*, was more abundant in normal colorectal tissues than in CRC tissues [51], while the others demonstrated that *genus Lactococcus* was enriched in CRC patients compared with healthy individuals [52].

Accumulating evidence suggests that gut microbiota are crucial drivers and potential therapeutic targets for DSCs. A series of clinical studies demonstrate that antibiotics (e.g., vancomycin), fecal microbiota transplantation, probiotics, and dietary interventions may be conducive to improving the efficacy of DSC therapies and reducing treatment complications [53]. For instance, fecal microbiota transplantation could reconstitute the gut microbiota and induce a relative increase in regulatory T cells within the colonic mucosa to relieve immune checkpoint inhibitor-associated colitis [54]. *Lactobacillus rhamnosus* GG is able to inhibit the proliferation of colon cancer cells and exerts antitumor activity by sensitizing cancer cells to 5-Fluorouracil and Irinotecan [55]. Oral administration of live *Lactobacillus rhamnosus* GG has also been found to increase tumor-infiltrating DCs and T cells and further augment the antitumor activity of anti-programmed cell death 1 immunotherapy [56]. Noteworthily, some bacterial taxa could enhance the antitumor effects of cancer treatments, while others could impair the efficacy of antitumor agents. Hence, it remains a research priority for us to achieve a better understanding of the specific gut microbes involved in DSCs and of the mechanisms underlying their action, which may be translated into possible clinical benefits for DSC patients in the future. Nevertheless, some limitations still existed in our study. Firstly, as our GWAS data on gut microbiota are from phylum to genus levels, we may have missed some specific bacteria that causally correlate to DSCs at a more specialized level such as the species or strain level. Secondly, all participants in the datasets we used were of European ethnicity, which limits the extrapolation of our findings to other ethnicities. Thirdly, the number of SNPs available for some microbial taxa is limited, which may have led to biased results.

## 6. Conclusions

We accomplished a comprehensive screening of gut microbiota implicated in DSCs and identified the causal relationship between several microbial taxa and DSCs using a bidirectional two-sample MR analysis. Our discoveries can provide more theoretical support for modulating the composition of gut microbiota to improve the treatment efficacy and promote the development of microbiota-based therapeutic strategies as well as microbial biomarkers for DSCs.

## Figures and Tables

**Table 1:** The GWAS datasets for DSCs.

GWAS_ID	Disease	Consortium	Sample Size	Case	Control	Population
ieu-b-4960	Oesophageal cancer	UK Biobank	372,756	740	372,016	European
finn-b-C3_STOMACH	GC	Finngen	218,792	633	218,159	European
finn-b-C3_SMALL_INTESTINE	Small intestine cancer	Finngen	218,792	252	218,540	European
finn-b-C3_COLORECTAL	CRC	Finngen	218,792	3022	215,770	European
ieu-a-822	Pancreatic cancer	PanScan1	3835	1896	1939	European
ieu-a-1057	Gallbladder cancer	NA	907	41	866	East Asian
finn-b-C3_LIVER_INTRAHEPATIC_BILE_DUCTS	Liver cancer	Finngen	218,792	304	218,488	European

DSCs, digestive system cancers; GC, gastric cancer; CRC, colorectal cancer.

**Table 2:** The significant causal effect of gut microbiota on DSCs calculated using the IVW method.

Outcome	Exposure	SNPs (n)	*p*-Val	OR	95%CI
GC	*family Bacteroaceae*	3	0.001	0.156	(0.053, 0.459)
	*family Enterobacteriaceae*	3	0.006	0.206	(0.067, 0.636)
	*genus Bacteroes*	3	0.001	0.156	(0.053, 0.459)
	*genus Eggerthella*	5	0.005	0.464	(0.270, 0.796)
	*genus Lachnospira*	1	0.026	0.079	(0.008, 0.739)
	*order Enterobacteriales*	3	0.006	0.206	(0.067, 0.636)
	*genus Escherichia Shigella*	4	0.025	3.099	(1.152, 8.338)
	*genus Eubacterium fissicatena group*	5	0.038	1.648	(1.028, 2.642)
	*genus Ruminococcaceae UCG014*	6	0.047	1.911	(1.010, 3.615)
Small intestine cancer	*family Clostriales vadin BB60 group*	10	0.030	0.422	(0.194, 0.918)
	*family Peptostreptococcaceae*	8	0.035	0.292	(0.093, 0.918)
	*genus Anaerofilum*	6	0.033	0.444	(0.211, 0.936)
	*genus Streptococcus*	8	0.045	0.266	(0.073, 0.969)
	*order Lactobacillales*	9	0.032	0.314	(0.109, 0.903)
	*genus Candatus Soleaferrea*	2	0.030	5.166	(1.171, 22.797)
CRC	*family Clostriales vadin BB60 group*	10	0.044	0.751	(0.569, 0.992)
	*genus Lachnospiraceae FCS020 group*	7	0.003	0.607	(0.439, 0.840)
	*phylum Euryarchaeota*	5	0.036	0.801	(0.650, 0.986)
	*phylum Proteobacteria*	4	0.037	0.613	(0.387, 0.971)
Pancreatic cancer	*genus Bilophila*	1	0.031	0.187	(0.041, 0.855)
	*genus Streptococcus*	2	0.032	0.253	(0.072, 0.888)
	*genus Ruminococcaceae UCG014*	1	0.029	6.101	(1.206, 30.873)
Liver cancer	*family Rhodospirillaceae*	7	0.014	0.357	(0.157, 0.809)
	*genus Escherichia Shigella*	4	0.026	0.198	(0.048, 0.821)
	*genus Eubacterium nodatum group*	3	0.009	0.382	(0.186, 0.783)
	*genus Family XIII AD3011 group*	7	0.030	0.261	(0.078, 0.876)
	*genus Turicibacter*	5	0.005	0.271	(0.109, 0.676)
	*genus Dorea*	5	0.010	8.102	(1.643, 39.965)
	*genus Lachnospiraceae UCG004*	7	0.039	3.199	(1.059, 9.662)
	*genus Oscillibacter*	12	0.021	2.129	(1.123, 4.035)
	*genus Paraprevotella*	9	0.032	1.961	(1.058, 3.636)

DSCs, digestive system cancers; IVW, inverse variance weighted; GC, gastric cancer; CRC, colorectal cancer.

**Table 3:** The significant causal effect of DSCs on gut microbiota calculated using the IVW method.

Exposure	Outcome	SNPs (n)	*p*-Val	Beta	95%CI
GC	*class Methanobacteria*	5	0.023	−0.087	(−0.161, −0.012)
	*family Methanobacteriaceae*	5	0.023	−0.087	(−0.161, −0.012)
	*family Oxalobacteraceae*	5	0.014	−0.076	(−0.137, −0.015)
	*genus Methanobrevibacter*	5	0.005	−0.109	(−0.184, −0.033)
	*genus Oxalobacter*	5	0.032	−0.070	(−0.134, −0.006)
	*order Methanobacteriales*	5	0.023	−0.087	(−0.161, −0.012)
	*phylum Euryarchaeota*	5	0.047	−0.074	(−0.147, −0.001)
	*genus Dialister*	5	0.001	0.070	(0.029, 0.110)
	*genus Eubacterium ventriosum group*	5	0.032	0.039	(0.003, 0.075)
Small intestine cancer	*genus Intestinibacter*	5	0.022	−0.033	(−0.060, −0.005)
	*genus Lachnoclostrium*	5	0.023	−0.024	(−0.044, −0.003)
	*genus Peptococcus*	5	0.004	−0.051	(−0.086, −0.016)
	*genus Collinsella*	5	4.80 × 10^-4^	0.040	(0.018, 0.063)
	*genus Erysipelotrichaceae UCG003*	1	0.046	0.057	(0.001, 0.113)
	*genus Eubacterium ruminantium group*	5	0.026	0.038	(0.005, 0.072)
	*genus Howardella*	4	0.002	0.085	(0.031, 0.138)
	*genus Lachnospiraceae UCG008*	5	0.002	0.051	(0.018, 0.084)
CRC	*genus Lactococcus*	9	0.004	−0.157	(−0.263, −0.051)
	*genus Marvinbryantia*	9	0.016	−0.081	(−0.148, −0.015)
	*genus Eggerthella*	9	0.045	0.100	(0.002, 0.197)
	*genus Eisenbergiella*	9	0.021	0.106	(0.016, 0.196)
Pancreatic cancer	*genus Eubacterium ruminantium group*	3	0.013	−0.104	(−0.186, −0.022)
	*genus Lachnospiraceae NC2004 group*	3	0.013	−0.116	(−0.208, −0.025)
	*genus Ruminococcaceae UCG014*	3	0.004	0.088	(0.028, 0.148)
Liver cancer	*genus Butyricimonas*	2	0.032	−0.056	(−0.107, −0.005)
	*genus Eubacterium nodatum group*	2	0.029	−0.104	(−0.197, −0.010)
	*family Actinomycetaceae*	2	0.027	0.071	(0.008, 0.135)
	*family Lactobacillaceae*	2	0.033	0.069	(0.006, 0.132)
	*genus Eubacterium brachy group*	2	0.044	0.087	(0.002, 0.171)
	*genus Lactobacillus*	2	0.036	0.068	(0.004, 0.132)
	*order Actinomycetales*	2	0.024	0.071	(0.009, 0.133)

DSCs, digestive system cancers; IVW, inverse variance weighted; GC, gastric cancer; CRC, colorectal cancer.

## Data Availability

The data are not publicly available due to privacy and ethical restrictions.

## Supplementary Materials

The following supporting information can be downloaded at: https://www.mdpi.com/article/10.3390/nu15132937/s1, Table S1: The strength of genetic instruments for exposures ensured by calculating the F statistic; Table S2: The details of sensitivity analyses for ten significant robust results that survived all the sensitivity analyses.

## Abbreviations

CIs: confidence intervals; CRC, colorectal cancer; DSCs, digestive system cancers; GC, gastric cancer; GWAS, genome-wide association study; IVs, instrumental variables; IVW, inverse variance weighted; MR, Mendelian Randomization; MR-PRESSO, MR-pleiotropy residual sum and outlier; OR, odds ratio; SCFAs, short-chain fatty acids; SD, standard deviation; SNPs, single nucleotide polymorphisms.
